# Reframing conservation physiology to be more inclusive, integrative, relevant and forward-looking: reflections and a horizon scan

**DOI:** 10.1093/conphys/coaa016

**Published:** 2020-04-04

**Authors:** Steven J Cooke, Christine L Madliger, Rebecca L Cramp, John Beardall, Gary Burness, Steven L Chown, Timothy D Clark, Ben Dantzer, Erick de la Barrera, Nann A Fangue, Craig E Franklin, Andrea Fuller, Lucy A Hawkes, Kevin R Hultine, Kathleen E Hunt, Oliver P Love, Heath A MacMillan, John W Mandelman, Felix C Mark, Lynn B Martin, Amy E M Newman, Adrienne B Nicotra, Sharon A Robinson, Yan Ropert-Coudert, Jodie L Rummer, Frank Seebacher, Anne E Todgham

**Affiliations:** 1 Fish Ecology and Conservation Physiology Laboratory, Department of Biology and Institute of Environmental and Interdisciplinary Science, Carleton University, 1125 Colonel By Dr., Ottawa, ON, K1S 5B6, Canada; 2 School of Biological Sciences, The University of Queensland, Brisbane, 4072, Australia; 3 School of Biological Sciences, Monash University, Clayton, Victoria 3800, Australia; 4 Department of Biology, Trent University, 1600 West Bank Drive, Peterborough, ON, K9L 0G2, Canada; 5 School of Life and Environmental Sciences, Deakin University, Geelong, Victoria 14 3216, Australia; 6 Department of Psychology, Department of Ecology & Evolutionary Biology, University of Michigan, Ann Arbor, MI 48109, USA; 7 Instituto de Investigaciones en Ecosistemas y Sustentabilidad, Universidad Nacional Autónoma de México, Antigua Carretera a Pátzcuaro 8701, Morelia, Michoacán, 58190, Mexico; 8 Department of Wildlife, Fish & Conservation Biology, University of California, Davis, One Shields Avenue, Davis, CA 95616, USA; 9 Brain Function Research Group, School of Physiology, University of the Witwatersrand, 7 York Rd, Parktown, 2193, South Africa; 10 College of Life and Environmental Sciences, Hatherly Laboratories, University of Exeter, Prince of Wales Road, Exeter, EX4 4PS, UK; 11 Department of Research, Conservation and Collections, Desert Botanical Garden, Phoenix, AZ 85008, USA; 12 Department of Biology, George Mason University, Fairfax, VA 22030, USA; 13 Department of Integrative Biology, University of Windsor, 401 Sunset Avenue, Windsor, ON N9B 3P4, Canada; 14 Department of Biology and Institute of Biochemistry, Carleton University, 1125 Colonel By Dr., Ottawa, ON K1S 5B6, Canada; 15 Anderson Cabot Center for Ocean Life, New England Aquarium, 1 Central Wharf, Boston, MA 02110, USA; 16 Department of Integrative Ecophysiology, Alfred Wegener Institute Helmholtz Center for Polar and Marine Research, Am Handelshafen 12, 27574 Bremerhaven, Germany; 17 Global Health and Infectious Disease Research, University of South Florida, 3720 Spectrum Boulevard, Tampa, FL 33612, USA; 18 Department of Integrative Biology, University of Guelph, Guelph, ON, N1G 2W1, Canada; 19 Research School of Biology, Australian National University, Canberra, ACT 2601, Australia; 20 School of Earth, Atmospheric and Life Sciences (SEALS) and Centre for Sustainable Ecosystem Solutions, University of Wollongong, Wollongong, NSW 2522, Australia; 21 Centre d'Etudes Biologiques de Chizé, CNRS UMR 7372 - La Rochelle Université, 79360 Villiers-en-Bois, France; 22 ARC Centre of Excellence for Coral Reef Studies, James Cook University, Townsville, QLD 5811, Australia; 23 School of Life and Environmental Sciences A08, University of Sydney, NSW 2006, Australia; 24 Department of Animal Science, University of California Davis, One Shields Ave. Davis, CA, 95616, USA

**Keywords:** conservation physiology, horizon scan, evidence, Sustainable Development Goals

## Abstract

Applying physiological tools, knowledge and concepts to understand conservation problems (i.e. conservation physiology) has become commonplace and confers an ability to understand mechanistic processes, develop predictive models and identify cause-and-effect relationships. Conservation physiology is making contributions to conservation solutions; the number of ‘success stories’ is growing, but there remain unexplored opportunities for which conservation physiology shows immense promise and has the potential to contribute to major advances in protecting and restoring biodiversity. Here, we consider how conservation physiology has evolved with a focus on reframing the discipline to be more inclusive and integrative. Using a ‘horizon scan’, we further explore ways in which conservation physiology can be more relevant to pressing conservation issues of today (e.g. addressing the Sustainable Development Goals; delivering science to support the UN Decade on Ecosystem Restoration), as well as more forward-looking to inform emerging issues and policies for tomorrow. Our horizon scan provides evidence that, as the discipline of conservation physiology continues to mature, it provides a wealth of opportunities to promote integration, inclusivity and forward-thinking goals that contribute to achieving conservation gains. To advance environmental management and ecosystem restoration, we need to ensure that the underlying science (such as that generated by conservation physiology) is relevant with accompanying messaging that is straightforward and accessible to end users.

## Introduction

Although physiological tools, concepts and knowledge have been applied to various conservation issues, environmental problems and resource management challenges for many decades (think back to Rachel Carson and Silent Spring in 1962), it has only been in the last two decades or so that this has been codified as a formal area of research—or even a discipline—‘conservation physiology’ (Wikelski and Cooke, 2006; Franklin & Seebacher 2012; Cooke *et al*., 2013). There has been a rapid maturation of the discipline with, in 2013, the initiation of a peer-reviewed journal titled ‘*Conservation Physiology*’ as well as the publication of a conceptual framework to help provide structure to research and its application to decision-making, practice and policy (Coristine *et al*., 2014). Several authors have mused about the challenges for delivering on the promise of conservation physiology (e.g. Cooke and O’Connor, 2010), yet there are also a growing number of success stories (Madliger *et al*., 2016) supported by an ever-expanding conservation physiology tool box (Madliger *et al*., 2018).

Conservation physiology is an applied discipline, and it is essential to re-assess frequently what is needed to ensure that the research community is generating the science required by knowledge users (e.g. resource managers, conservation practitioners) and decision makers (e.g. policymakers, politicians, funders). If the knowledge being generated is not relevant to end users and does not inform the major environmental and conservation problems of today, then it is failing. To that end, taking time to reflect on the discipline while at the same time considering opportunities for growth and refinement will be profitable. As a diverse group of researchers whose work spans taxa (from plants to mammals), realms (from the skies to the depths of the oceans), regions (from five continents but with a global research footprint), and subdisciplines/tools (from physiological genomics to bio-logging), we bring toge-ther our collective perspectives to engage in both a reflective and forward-looking exercise. First, we consider how conservation physiology can be more inclusive and integrated to ensure that it has the potential to have the greatest impact on policy and practice. Second, we engage in a horizon scan to identify the ways in which conservation physiology can be used to address persistent or emerging challenges (and opportunities) that will ensure we remain attuned to the conservation challenges of today. Our intention is to continue to advance conservation physiology as a mission-oriented discipline with meaningful impact that will help to mitigate declines in biodiversity and enable sustainable management of natural resources, thereby contributing to the realization of the Sustainable Development Goals adopted by all United Nations Member States in 2015 as a universal call to action to protect the planet and support human well-being.

## The evolution of conservation physiology to be a more integrative and inclusive discipline

Conservation physiology is an inherently integrative discipline, involving the application of field- and/or laboratory-based individual assessment tools to conservation problems that can span populations, and geographic and temporal scales (Cooke *et al*., 2013a). Even over the past 14 years since the first formal description of the discipline was published (Wikelski and Cooke, 2006), evidence has accumulated that the refinement of the definition of conservation physiology has led to expanding its reach (Cooke *et al*., 2013a). (For the purpose of this paper, we consider ‘integrative’ to mean the ways in which conservation physiology brings together different tools, concepts and knowledge, often arising from different physiological sub-disciplines but also allied disciplines (like behavioural ecology, trophic ecology and veteri- nary medicine). It also includes the idea that conservation physiology is an applied discipline embedded in the broader mission-oriented discipline of conservation science. To thatend, ‘integrative’ also means fully considering the policy implications of findings.) Part of this growth originates from the acknowledgement that some of the ‘simplest’ physiological measurements, such as gross metrics of body condition or mass, can provide valuable information on whole-organismal physiological function (see definition of ‘conservation physiology’ in Cooke *et al*., 2013a). Indeed, this broad definition of physiology can allow the discipline’s toolbox to benefit species that are difficult to access or repeatedly sample. For example, teams of conservation physiologists rely on photographs of right whales (*Eubalaena glacialis* and *E. australis*) to assess health and condition (Hamilton *et al*., 2007; Frasier *et al*., 2009; North Atlantic Right Whale Consortium, 2019). These images reflect body condition and nutritional state (e.g. fuel stores), reproductive status and exposure to anthropogenic stressors (Rolland *et al*., 2016; Pettis *et al*., 2017; Christiansen *et al*., 2018), making them a highly accessible, non-invasive, physiological assessment tool. With the advent of increasingly less invasive tools (including many more ‘traditional’ metrics, such as hormone levels), there is also great potential for conservation physiology to encompass new species/taxa and conservation contexts, further expanding the discipline into new integrative territories.

Additionally, many conservation scientists are advocating proactive approaches that reverse disturbances early, well in advance of when a species would be listed as Threatened/Endangered or experience a potentially irreversible decline (e.g. Drechsler *et al*., 2011; Benson, 2012; Donlan, 2015). This more proactive approach to conservation science creates opportunities for conservation physiology, as physiological changes often occur quickly and with great sensitivity, providing early-warning signals (Ellis *et al*., 2012; Cooke *et al*., 2013a). A progressive form of conservation physiology also recognizes that integrating additional disciplines are necessary to solve almost any conservation problem. For example, physiology and behaviour are tightly linked, and ignoring the behavioural consequences of physiological changes risks missing key information on when, where and how to address disturbances (Cooke *et al*., 2013b). These types of fruitful integrations can be more easily identified if we view conservation physiology as a study of the mechanisms underlying species responses to changes in their environment (Franklin and Seebacher, 2012). By understanding how variation in physiological traits occurs over different spatial and temporal scales, individual physiological metrics can be used to predict landscape-level ecological outcomes (i.e. macrophysiology; Chown and Gaston, 2016). Efforts to link the physiological variation among individuals, populations, landscapes and macroecological processes across various spatial and temporal scales will help to make conservation physiology more relevant to practitioners (Cooke *et al*., 2014; Chown and Gaston, 2016). Indeed, we are slowly gaining more evidence that the often-cited challenge of linking individuals to populations (summarized in Cooke and O’Connor 2010) is surmountable (Bergman *et al*., 2019) and that conservation physiology does provide information that can be used to manage and restore populations and ecosystems (Madliger *et al.,* 2016).

Beyond integration with other disciplines in the natural sciences, every conservation issue can be viewed as being set within a social context (Hirsch *et al*., 2011). Finding a solution involves more than just designing a management scenario based on ecological or physiological observations or experimentation; the successful application of a management technique will be influenced by local human perceptions, politics and economics, among other social structures (Fischer *et al*., 2009; Hirsch *et al*., 2011; Kaplan-Hallam and Bennett 2018). This interface also highlights how the ongoing evolution of conservation physiology to become more integrative should be paired with efforts to enhance inclusivity. For the purpose of this paper, we consider inclusive to mean that the way we do conservation physiology work can be contextualized around the recognition that diverse perspectives and different ways of knowing collectively improve our ability to solve conservation problems. As the discipline matures, this also means being proactive in ensuring that our community of scholars is diverse in all forms and that we work to build capacity in regions where there is interest in or need for conservation physiology. In other words, we want to have a welcoming and broad community of research and practice. In many cases, researchers and practitioners may not be formally trained in either physiology or conservation science, increasing the need for collaborative and cross-disciplinary relationships. Diverse voices from policymakers and practitioners can lead to better-designed research that directly addresses on-the-ground concerns (Dilling and Lemos, 2011; Meadow *et al*., 2015), incorporates local knowledge and experiences (Fazey *et al*., 2006; Cvitanovic *et al*., 2016) and develops solutions with known constraints for implementation in mind from inception (Cvitanovic *et al*., 2015; Cvitanovic *et al*., 2019). Geographic inequality of conservation physiology research may also be hindering the field. For example, the majority of *Conservation Physiology*’s publications are by authors from high- or upper-middle-income countries and/or concern work completed in those countries (Steven Cooke, *pers obs*), but much of the world’s most biodiverse and imperilled regions are located in more marginalized countries.

## Horizon scan—preamble

Horizon scanning is now a well-established approach to identify emerging issues and opportunities. In the context of environmental issues, horizon scanning can help to direct research activities (and funding) and accelerate progress (Sutherland and Woodroof, 2009). There is no single formula that can be used to conduct a horizon scan, but there are some best practices. First and foremost, it is key to have a diverse team working at the frontiers of a discipline (or ideally across disciplines). This team should be well-read about the key challenges and basic knowledge around a conservation issue, and for an applied environmental scan, they should also engage routinely with practitioners and decision-makers to understand their information needs. To that end, the current team of Editorial Board members for the journal *Conservation Physiology* (along with two early career scholars in this realm; i.e. Madliger and Cramp) were recruited to participate in this activity. We collaboratively generated a list of topics for the horizon scan and then small teams of two to four individuals crafted each section. All co-authors then had the opportunity to edit and refine those sections. Here, we present what we believe are emerging issues and opportunities for which conservation physiology has the potential to make a significant impact. We have tried to provide reasonably equal (but brief) coverage for all of the topics except for the last one—on the Sustainable Development Goals (SDGs). Given the global importance of the SDGs and the interesting ways that conservation physiology could contribute to addressing them, we intentionally explore that topic in greater depth using a table.

### 

#### Informing the decade of ecosystem restoration

In March of 2019, the United Nations launched the ‘Decade of Ecosystem Restoration 2021–2030’ in recognition of the manifold negative effects that humans have had on the planet, but also a realization that there is an opportunity for restoration (but see Cooke *et al*., 2019). Restoration science and practice have evolved in recognition of the need to learn from successes and failures (through monitoring and adaptive management); yet, it remains imperfect, and in many cases, restoration occurs without any formal monitoring (Wortley *et al*., 2013). Physiology informs restoration in a number of ways (reviewed in Cooke and Suski, 2008). For example, physiology can be used to diagnose a problem (e.g. to reveal that an ecosystem or its constituent biotic parts are threatened) and identify specific threats that led to a degraded state, thus allowing such stressors to be addressed prior to embarking on restoration initiatives. In this way, it can also identify priority sites for restoration as has been done for coral reefs using molecular techniques (Ammar *et al*., 2000). Physiology can also be used to monitor outcomes and determine the success of an existing or newly implemented restoration program, often directing future actions. It is also possible to identify candidate species (or populations) that will do well in degraded sites and can thus be used for restoration purposes (e.g. often done in the context of wetland restoration with tolerant plant species; Pywell *et al*., 2003). What is particularly useful with physiology is that the traits that are measured are often ones that respond more quickly than traditional attempts to assess changes in population size and/or community structure (e.g. Adams and Ham, 2011). In doing so, physiology allows one to focus not only on the structural aspects of restoration but also on the functional aspects that are often difficult to assess (Herrick *et al*., 2006). Physiology therefore has the potential to deliver great advances during the Decade of Restoration by providing objective tools that directly inform restoration actions. More work is needed to demonstrate the value of physiology for restoration by highlighting success stories and by working more closely with restoration practitioners to promote knowledge sharing.

#### Achieving urban renewal and ecological harmony

Urbanization is considered one of the most prominent threats to the natural world, with 55% of the world’s population residing in urban areas today and projected increase to 68% by 2050 (United Nations, 2018). This human invasion into the natural landscape yields an entirely novel ecosystem for local wildlife (Shochat *et al*., 2006; Møller, 2009; Birnie-Gauvin *et al*., 2016; Alberti *et al*., 2017; Ouyang *et al*., 2018; Rivkin *et al*., 2019), and organisms inhabiting these environments are subject to a suite of novel stressors and selective pressures. There is dire need for us to rethink what is needed to achieve urban renewal that is harmonious with nature (e.g. spaces for biodiversity in the city at scales that maintain ecosystem function). In the city, wildlife are exposed to the hazardous by-products of anthropogenic activity, such as pedestrian traffic, vehicular collisions, pollution (Haynes *et al*., 2019) and artificial light and noise (Ashley and Robinson, 1996; Robert *et al*., 2015; Gaston, 2018). Additionally, through the exclusion of native predators and the introduction of exotic species and novel food resources, human activity has restructured ecological communities across the globe (Valcarcel and Fernandez-Juricic, 2009; Fischer *et al*., 2012; Blancher, 2013). Nonetheless, in part due to phenotypic adjustments, some species can exploit these evolutionarily novel conditions and thrive with population densities greatly exceeding those of their conspecifics in natural landscapes (Prange *et al*., 2003; Kark *et al*., 2007; Parker and Nilon, 2012; McDonnell and Hahs, 2015). These adjustments may, in part, be underpinned by physiology (Bonier 2012; Killen *et al*., 2013). Conservation physiology represents a powerful integration of proximate tools and ultimate scaffolds that can be employed to first describe and then probe phenotypic divergence among environments before assessing the success of environmental mitigation in urban areas and fine-tuning ecological initiatives. Importantly, the holistic approach of conservation physiology allows for sophisticated study of novel regulatory processes in the complex web of urban wildlife communities from either top down (e.g. predation) or bottom up (e.g. food subsidies) perspectives to examine the persistence of both conserved physiological regulation and emergence of novel physiological phenotypes across scales, from molecular to community levels.

#### Rewilding for impact

Although its definition is debated (Pettorelli *et al*., 2018; Hayward *et al*., 2019), rewilding includes diverse approaches aimed at restoring wildness and ecological function (Perino *et al*., 2019). There is widespread interest in the concept, given current threats to biodiversity. Yet, there are challenges associated with implementing rewilding projects, particularly within the framework of current legislation and land-use policies, and there is also a lack of empirical evidence to examine the risks and benefits of rewilding (Pettorelli *et al*., 2018). The development of sound rewilding plans requires an understanding of the causal mechanisms underlying previous loss of species from an ecosystem, as well as insights on the functioning of reintroduced species to modified environments (Cooke and Suski, 2008). There is also a need to assess the acute stressors associated with the process of conservation translocations, including monitoring and improving the physiological welfare of organisms during and immediately after relocation (Tarszisz *et al*., 2014). Physiological tools provide a clear path forward to investigate these mechanisms; for example, the advent of animal-borne digital bio-logging devices has enabled physiologists to measure markers of stress and health in free-living animals in natural and modified habitats (Wilson *et al*., 2014; Wilson *et al*., 2015). The rapid rate at which environments are changing makes classical management approaches unsuitable for planning and assessing rewilding interventions. The use of eco-indicating or umbrella species in remote ecosystems illustrates this issue well: population monitoring needs to be conducted over several decades to deliver trends of change (Post, 2004) while signals of change from finer-scale behavioural markers may be blurred by individual plasticity. Physiological markers offer the dual advantage of often being honest signals and responding at different time scales, from extremely rapid changes in baseline glucocorticoid levels (e.g. Ricklefs and Wikelski, 2002) to integrative markers of the accumulated level of stress endured by an aging organism (e.g. telomere loss, Blackburn and Epel, 2012). Recent advances in portable laboratory methodologies now provide a panel of physiological tools that can be explored to provide rapid insights on the success of rewilding projects which will presumably become more important with a growing number of ex situ breeding programs (see next section).

#### Captive breeding for success

With continued loss of biodiversity, there is increasing reliance on captive breeding programs to supplement wild populations. However, within captive environments there exists substantial variation in breeding success among individuals, populations and species, which is often attributed to captivity-induced stress (Mason, 2010). Conservation physiology continues to play an important role in understanding the causes of a breeding failure by providing mechanistic understanding of links between stress and reproduction (Dickens and Bentley, 2014) and by providing the tools necessary for proactive monitoring (Madliger *et al*., 2018). For example, in addition to routine veterinary panels to assess health, variation among individuals in stress responsiveness could be assessed upon first capture, which could implicate individuals most likely to adjust successfully to captivity (Dickens *et al*., 2009; Angelier *et al.,* 2016). Endocrine studies to understand the reproductive physiology of rhinoceros species proved essential for identifying effective captive breeding programs (Roth, 2006), while amphibian endocrine studies (Silla and Byrne, 2019) and studies of the nutritional and digestive physiology and environmental tolerances of early life stages of imperilled anurans facilitated husbandry success in captivity (Pryor, 2014). Similar research in the plant realm has allowed for refinements in storage and eventual germination of seed germplasm (Fu *et al*., 2015). Moreover, the captive breeding of plants provides an opportunity for selecting individuals/provenances for assisted migration by linking physiological traits and environmental associations using evolutionary perspectives and genomic tools (e.g. Supple *et al*., 2018). When dealing with threatened species, there has been a general reluctance to incorporate some physiological techniques, particularly for vertebrates. These techniques are perceived as invasive and thus potentially counter to conservation aims. The continued development of non-invasive physiological methods (e.g. Madliger *et al*., 2018) will hopefully assuage some of these concerns, allowing physiological indices of health and performance (particularly related to reproduction/germination and growth) to assume a more predictive role in captive breeding programs for plants and wildlife.

#### Siting and monitoring protected areas

Protected areas, whether on land (Hansen and DeFries 2007) or sea (Agardy, 1994), are increasingly being recognized as effective conservation options. In fact, there are now efforts to expand protected areas such that they cover up to 20% of the globe (https://www.iucn.org/theme/protected-areas/about/iucn-global-protected-areas-programme;https://www.cbd.int/aichi-targets/target/11;Watson *et al*., 2014). Yet, challenges remain with both determining which areas should be protected (i.e. siting) as well as evaluating the effectiveness of protected areas in achieving various conservation outcomes (Thiel *et al*., 2008, 2010). Physiological tools can be used to identify the extent to which organisms in a given region at a given time are subject to stressors, which can be used to distinguish more ‘pristine’ from degraded sites. For example, physiological and fitness-related metrics (e.g. growth, survival stress indicators) were used to evaluate the effectiveness of restored floodplain habitat set aside for rearing pre-out migrating salmon smolts in California’s central valley to bolster imperilled salmon populations (Jeffres *et al*., 2008). Physiological parameters (e.g. thermal sensitivity) also often mediate species survival in altered landscapes (Nowakowski *et al*., 2018) and can be integrated into predictive climate change models to assist in the prioritization of suitable restoration habitat for critically endangered species (Brown *et al*., 2016). Once a protected area has been established, physiological tools can be used to assess ecosystem function and the health and condition of resident organisms. For instance, measurements of nitrogen content and δ^15^N in plant tissue (Díaz-Álvarez *et al*., 2018), which can be further related to hyperspectral remote sensing signatures (Garbulsky *et al*., 2011), can help estimate the level and origin of atmospheric pollution, a major leading cause of global biodiversity loss following changes in land use and climate (Rockström *et al*., 2009). Future physiological research for monitoring natural protected areas could focus on developing tools for early warning and intervention when multiple environmental stressors lead to forest die off (McDowell *et al*., 2008) or for controlling invasive species proliferation (Vilá *et al*., 2011). If such efforts involve local communities and consider other ways of knowing (e.g. Indigenous knowledge holders), they could improve the ecological success and gain political favour by decision-makers, an increasingly necessary condition for the successful persistence of natural protected areas (Watson *et al*., 2014).

#### Mitigating big infrastructure projects

Governments are increasingly realizing that prioritizing ecosystem functioning can balance human satisfaction, wildlife health and economic return (Semeniuk *et al*., 2009a). However, in the modern era of increasingly short turnaround times, rapid administrative turnover and highly scrutinized budgets, the effective management of big infrastructure projects (e.g. dams, mines, nuclear power plants, highways) requires rapid and reliable predictors of ecosystem state. Because physiology sits at the heart of organismal function, it is increasingly valued for its ability to link environmental variation with organismal performance and fitness (i.e. reproduction and survival; Madliger *et al*., 2016). As such, conservation physiology can help developers, governments and environmental assessors maximize efficiency by providing valuable tools that integrate the planning and implementation of infrastructure projects with an assessment of the ecosystem impact of the work (Madliger *et al*., 2017, 2018). Physiological tools have already been used to assess human-induced impacts on organisms (e.g. Semeniuk *et al*., 2007, 2009b; Crino *et al*., 2013; Kleist *et al*., 2018), highlighting that future projects can benefit from this type of integrative planning. In an ideal scenario, physiological tools are brought in at the planning stages to set baseline standards for ‘normalcy’ before work even begins. Alternatively, given that the impacts of exploratory works are also substantial and often unregulated, periodic measurements of physiological performance at these preliminary stages can help dimension the actual mitigation actions that will be required (Ellis *et al*., 2012). Using previous research on how and why environmental change impacts these traits, acceptable standards of change can then be agreed upon before projects begin (Blickley *et al*., 2012; Patricelli *et al*., 2013). When physiological traits with known relationships to performance and fitness are used (e.g. glucocorticoids, Bonier *et al*., 2009; Sorenson *et al*., 2017; photosynthetic efficiency (Fv/Fm) French *et al*., 2017), monitoring physiological changes or responses at multiple points in the project implementation process allows partners to determine whether thresholds of acceptable function (e.g. high intensity swimming at a dam site that contribute to migration failure later on in the form of a carryover effect; Burnett *et al*., 2014) have been surpassed at which point projects can be halted or altered. By quantifying changes in physiological function from before to after a project is undertaken, partners can assess the degree of success of their project mitigation procedures. Because not all individuals respond to stressors in the same way (Cockrem, 2013; Madliger and Love, 2014), this information can also be used to scale individual responses up to predict impacts on the local affected population. Ultimately, the team should be able to use this integrated set of information to better design future projects that further reduce (or even eliminate) impacts on populations, specific species of concern and whole ecosystems.

#### Tackling emerging pollutants

Investigating the physiological effects of pollutants and toxicants (e.g. heavy metals, petrochemicals, xenohormones) across taxa has provided researchers a foundation on which to investigate emerging stressors (e.g. microplastics, anthropogenic noise and artificial light) as well as led to developments in nature-based technology to clean up habitats (i.e. bioremediation; Choudhary *et al*., 2017) and even nature-based substitutes to reduce such stressors (e.g. bioplastics; Huang and Daboussi, 2017). The most prominent and likely to be long-lasting emerging stressor—microplastics (plastics <5 mm, including nanoplastics <0.1 μm)—has been cited as one of the most relevant topics for global conservation in the 21st century (Barnes *et al*., 2009). Research on this topic is in its infancy, and the extent to which there are physiological consequences of microplastics remains unclear. Anthropogenic noise represents yet another ‘emerging stressor’ but has actually been studied in the context of marine life since the 1970s, with an early focus on echolocating marine mammals (Payne and Webb, 1971), and the body of work has grown substantially in recent years (e.g. Popper and Hastings, 2009; Morley *et al*., 2014; Williams *et al*. 2015). There are many anthropogenic sources of terrestrial (e.g. urban, transportation, industrial activity, military aircraft) and underwater (e.g. sonar, pile-driving, seismic testing and renewable energy devices, with motorized vessels being the most pervasive) noise (Popper and Hastings, 2009). Artificial light at night (ALAN) or ‘light pollution’ is experienced by >80% of the world’s human population (Falchi *et al*., 2016), so it is not surprising that ALAN has been linked to negative impacts on a wide array of aquatic and terrestrial biota. Most attention has focused on birds and mammals with recent attention toward fishes and other aquatic life, with metabolic, oxidative and immune stress, reproductive failure and altered growth rates being the most pervasive physiological responses (e.g. Bedrosian *et al*., 2011; Gaston *et al*., 2015; O’Connor *et al*., 2019). For the aforementioned emerging stressors and others not discussed, mitigation strategies must be part of the conversation and included in the experimental design of conservation physiology studies. It is no longer enough to report that anthropogenic noise and artificial light negatively impact aquatic and terrestrial organisms. Research needs to be directed toward testing whether/how these stressors can be abated effectively with no ill-effects on wildlife. Furthermore, with GIS, satellite and other technologies, calculations can be made (e.g. annual input of plastics into the oceans from the top polluting river, the Yangtze in China, is 333 000 tonnes; Lebreton *et al*., 2017), maps can be created (e.g. The New World Atlas of Artificial Night Sky Brightness; Falchi *et al*., 2016), and models can be built (e.g. continental-scale sound models to predict the effects of anthropogenic noise in protected habitats; Buxton *et al*., 2017) to understand how spatial and temporal patterns of these emerging stressors might threaten wildlife at multiple scales.

#### Predicting climate chaos consequences

The global climate is changing rapidly as a result of human activity. Increasing temperatures are the best-described phenomenon of climate change, but by far not the only one (van de Pol *et al*., 2017). In addition to causing increasing temperatures, dissolved CO2 contributes to decreasing pH. Acidification and warming together have a negative, interactive impact on biomaterials such as byssal threads of mussels and calcification rates of invertebrate skeletons and shells (Carrington *et al*., 2015). These effects are compounded by increasing storm and wave action and, together, warming, acidification and storms have caused the decline of massive areas of coral reef, for example (Hughes *et al*., 2017). Beyond coral reefs, climate change-induced altered wind and ocean circulation patterns interfere with animal migrations (Fenkes *et al*., 2016; Nourani *et al*., 2017). Warming is also not a uniform process, and the average increase in temperature across the globe is associated with varied impacts—increasing and decreasing precipitation regimes and increased frequency of extreme thermal and precipitation events. Such interacting environmental drivers cause unpredictable climate scenarios (‘chaos’) that impact biogeography and biodiversity as a result of altered movement patterns, extirpations of species from areas that have become climatically unsuitable, and changes in community dynamics due to differential sensitivity and responses of species to change (Pecl *et al*., 2017). The potential to reach tipping points in community composition and function (Harris *et al*., 2018) will in part be determined by physiological tolerances of the components of those communities. For example, on the Antarctic continent, retreating snow banks, lower summer temperatures, increased winds and increased evapotranspiration are making water less biologically available during the growing season leading to declining health of the dominant moss plants (Robinson *et al*., 2018). The additive stress of these extreme events superimposed on the longer-term drying trend has resulted in species turnover with the Antarctic endemic, *Schistidium antarctici*, being outcompeted by two cosmopolitan species (Pecl *et al*., 2017; Robinson *et al*., 2018). Early warning of this change comes from physiological tools (stable isotope and stress pigment changes) which are increasingly being linked to remote sensing technologies (Malenovsky *et al*., 2017). Conservation physiology is therefore an essential tool to predict the effects of changing and interacting environmental drivers on performance, and therefore persistence and distribution of organisms (Wikelski and Cooke, 2006). Its strength lies in the mechanistic approach it takes to understand climate impacts, which moves well beyond the correlational descriptions of changing patterns to include mechanistic laboratory and field studies. Predictions that incorporate experimental evaluation of realistic complexity and stochasticity of multiple interacting stressors in complex systems are difficult, but essential (Helmuth *et al*., 2014).

#### Moving beyond monitoring toward control of emerging pathogens and disease

Infectious diseases rank as major challenges for wildlife management, with new diseases emerging at an alarming rate (Daszak *et al*., 2001, Jones *et al*., 2008). Current responses are biased toward surveillance of ‘target’ pathogens (Hill-Cawthorne and Sorrell, 2016) or exposure thereof (Plowright *et al*., 2019) and tend to be slow (Hill-Cawthorne and Sorrell, 2016). By contrast, new frameworks derived from conservation physiology can identify impactful species (Han *et al*., 2015, Downs *et al*., 2019), populations (Gervasi *et al*., 2015), individuals (Martin *et al*., 2019) or even sites (Paull *et al*., 2011). Indeed, these perspectives might enable us to predict, instead of respond to, risk of disease spread or spillover (Becker *et al*., 2019b). Central to most of this research is the concept of host competence, the propensity of a host to generate new infections (Martin *et al*., 2016). Particular traits have been implicated as the drivers of variation in within and between host competence (Adelman and Hawley 2017); parasite tolerance in particular seems important (Binning *et al*., 2017; Burgan *et al*., 2018), but it is notoriously hard to measure in wildlife (Burgan *et al*., 2019). Fortunately, some physiological proxies of tolerance, such as glucocorticoid concentrations (Gervasi *et al*., 2017) and immune gene expression from circulating blood cells (Adelman *et al*., 2013; Jackson *et al*., 2014; Burgan *et al*., 2019), have been identified. However, whereas simple blood- or faeces-borne indices are widely touted as useful proxies (Besson and Cree, 2011; Tarszisz *et al*., 2014), there are many shortfalls or outright failures (Sorenson *et al*., 2017; Martin *et al*., 2018). Although we have long thought that chronic stress predisposes wildlife to infection (Hing *et al*., 2016), we need to move beyond surveys of faecal glucocorticoid metabolites, parasite burden and other indirect measures of health to more direct efforts such as gene expression from important tissues. Another major challenge is shifting the research bias from vertebrates, particularly mammals and birds (Altizer *et al*., 2018), to other vertebrates, invertebrates generally (Adamo and Parsons, 2006) and particularly vectors (Mordecai *et al*., 2017). Also, further work is needed to link individual-scale metrics to landscape-scale processes (Becker *et al*., 2019a), which will help us develop realistic, but still simple, epidemiological models that better predict infection dynamics in nature (Downs *et al*., 2014).

#### Strengthening and mining the evidence base

In civil society, evidence should guide decision-making and be the basis for policy. This is certainly the case for issues related to the environment and is consistent with the ‘evidence-based conservation and environmental management’ movement (Sutherland *et al*., 2004). In the scientific domain, research often comes in the form of peer-reviewed papers or technical reports. Yet, just because something is published in a reputable peer-reviewed journal (even one with a high impact factor) does not mean that it is necessarily of high quality. Indeed, the premise of evidence-based conservation is assembling all possible knowledge on a topic (to ensure no bias) in a structured and repeatable manner and then evaluating its quality using a critical appraisal tool (and recording the basis for decisions regarding quality to ensure transparency) to eliminate the biased knowledge followed by generating a synthesis using the best available data. The most common means of doing so is through a systematic review (Pullin and Stewart, 2006), but there are also other forms of evidence synthesis that are used to inform decisions (Dicks *et al*., 2014). A burden of evidence built upon a foundation of high-quality empirical studies, as achieved through the Cochrane Reviews for human health care and policy, can bring the certainty needed for decision-makers and practitioners to ‘act’. Yet, if the research conducted by the conservation physiology community is deemed to be of low quality, it will not have the impact it intends to. Aspects of quality that are particularly important include having adequate replication (and avoiding pseudoreplication), use of appropriate controls, having robust sample sizes for both sexes and addressing various forms of bias. Cooke *et al*. (2017) provided guidance to the conservation physiology community on how to avoid these pitfalls. Another opportunity for the conservation physiology community is to take a leadership role in conducting systematic reviews (to a high standard—such as by following the guidelines of the Collaboration for Environmental Evidence). Indeed, researchers working on conservation behaviour recently published a coordinated suite of systematic maps (the precursor to a systematic review). There is opportunity for the conservation physiology community to do the same—tackling questions such as ‘Do alterations in physiological function in animals arising from human disturbance translate to declines in population size?’, ‘Do microplastics affect animal function?’ and ‘What are the determinants of native plant survival when used in ecological restoration?’.

#### Bending the curve for biodiversity

Biodiversity loss is accelerating (McGill *et al*., 2015) with collateral consequences for humanity (Diaz *et al*., 2006; Cardinale *et al*., 2012). Discussions surrounding biodiversity have extended to consider that we may be entering the sixth mass extinction (Ceballos *et al*., 2015). Given the threats to biodiversity, there have been a variety of initiatives intended to begin the task of arresting or even reversing the decline. Given the (accelerating) curve of biodiversity loss plotted over the past century, some have suggested we need to ‘bend the curve’ (Mace *et al*., 2018). Unfortunately, despite both global, top-down initiatives and local bottom-up initiatives, there is little evidence that we have made much progress toward meeting various international biodiversity targets (Tittensor *et al*., 2014). Conservation physiology has the potential to be used to set targets and monitor progress toward achieving them. Although increases in population size are the primary desirable outcome, conservation physiology can identify the drivers of decline and thus identify opportunities for threat mitigation. Indeed, to bend the curve(s) it will be necessary to identify organism-specific physiological levers that need to be addressed, as outlined for terrestrial animal species in Leclere *et al*. (2018). Moreover, while indicators are a key component of progress, the current suite of threat indicators all focus on population-level trends. Conservation physiology could be used to define other indicators that could be correlated with population trends while simultaneously enabling one to track responses to the key stressors (and thus the extent to which stressor mitigation is working). A number of organizations (e.g. WWF) are aligning efforts to focus on bending the curve such that this will be a profitable area of action in the coming years.

**Table 1:** **Conservation physiology and the UN Sustainable Development Goals.** Relevant conservation physiology research with potential to generate outcomes or benefits that are relevant to the UN Sustainable Development Goals

**Sustainable development goal**	**Physiological research**	**Outcomes/benefits**	**Example sources**
1. No poverty^1^	NA	NA	NA
2. Zero hunger	Use of metabolic rate estimates in the context of expected climate change to show how and where crop losses owing to herbivory by insects will be greatest: largely in temperate regions	Better forecasts of intervention requirements	Dillon *et al*. 2010; Deutsch *et al*., 2018
3. Good health and well-being	A wide range of research on the responses of vectors, and the diseases they carry, to climate change and other environmental disturbances	Enhanced understanding of disease risk now and into the future to guide interventions	Sternberg and Thomas 2014; Franklinos *et al*. 2019
4. Quality education	Citizen science in a variety of areas. For example, demonstrating changes to snail shell albedo in response to complex habitat changes	Engagement of citizens in science to benefit themselves and society	Silvertown *et al*. 2011; see also English *et al*. 2018
5. Gender equity	Demonstrations across the physiological sciences that considerations of gender are important in every field	Appreciation for the diversity of responses and sex-related traits across all of biological diversity	Millington and Rideout 2018; Candolin and Wong 2019
6. Clean water and sanitation	Pharmaceutical impacts on the physiology of aquatic species and populations with wider implications for biodiversity and for human health	Improvement of sanitation systems to remove plastic and pharma pollutants from grey-, black- and sto water streams	Wright-Walters and Volz 2009; Brooks 2018
7. Affordable and clean energy	Effects of alternative or more efficient energy sources on animal and human physiology demonstrated	Information can be used to manage implementation of alternatives so ensuring that affordable and clean energy has minimal biodiversity impacts	Gaston 2018; Thanker *et al*. 2018
8. Decent work and economic growth	Demonstrations of heat load for humans and domestic animals and consequences for decent work and economic success under changing climates	Development and adaptation options for areas forecast to be most affected by thermal extremes under future scenarios	Dunne *et al*. 2013; Oleson *et al*. 2013
9. Industry, innovation and infrastructure	Biomimetic and biotechnological applications arising from responses of animals and plants to the external environment—including antifreeze proteins and nanostructured surfaces	New innovations for industry which may revolutionize opportunities and improve efficiency	Watson *et al*. 2017; Mangiagalli *et al*. 2020
10. Reduced inequalities	No specific studies, but general demonstrations of the need to consider access inequality in education given the location of universities in cities	Opportunities as educators to improve opportunities for all those interested in conservation physiology	Weiss *et al*. 2018; White and Lee 2019
11. Sustainable cities and communities	Impacts of, and population and community responses to, urban heat islands and artificial night time lighting	Adaptation in cities to reduce biodiversity impacts while making cities more liveable	Gaston *et al*. 2013; Diamond *et al*. 2018
12. Responsible consumption and production	The geometric framework for nutrition, identifying the mechanistic basis of complex factors underlying the human obesity pandemic	Solutions for a growing global human health challenge, founded on first order physiological principles applicable generally	Simpson and Raubenheimer 2012; Leulier *et al*. 2017
13. Climate action	A broad range of research at the local, landscape, regional and global scales demonstrating the impacts of environmental change and elucidating their mechanistic basis	Empirical and theoretical support for the benefits of mitigation and for guiding adaptation given the climate change to which systems are already committed	Chown and Gaston 2008; Huey and Kingsolver 2019
14. Life below water	Demonstrations of increased threat to species and populations under climate warming and ocean acidification	Uptake of information into the IPCC Special Report on the Ocean and Cryosphere and availability of information for IPCC AR6 so focussing policy on mitigation	Stillman and Paganini 2015; Pinsky *et al*. 2019
15. Life on land	Limited warming tolerance of tropical terrestrial species, but better tolerance of invasive alien species than their indigenous counterparts	Uptake of original work into the IPCC Assessment Reports showing how vulnerable tropical environments are; further support for biosecurity interventions to prevent introduction of non-indigenous species	Deutsch *et al*. 2008; Janion-Scheepers *et al*. 2018
16. Peace, justice and strong institutions	Trait databases employing FAIR principles becoming available for an increasing diversity of physiological and closely related life history traits	Macrophysiological, meta-analytical and systematic reviews for evidence to investigate given pathways can be more readily undertaken to support informed decision-making	Kattge *et al*. 2011; Bennett *et al*. 2018
17. Partnerships	Multi-, cross- and interdisciplinary approaches to physiological research including those that build capacity and lead to solutions that work	Partnerships which provide information to address the challenges of the SDGs	Lapointe *et al*. 2018

^1^Alleviation of poverty requires concerted efforts of institutions and society more broadly. There is recognition that pursuing socially-oriented goals (like SDG 1) may lead to higher environmental impacts (Scherer *et al*., 2018) so there are indirect ways in which conservation physiology could be relevant.

#### Delivering on the SDGs

The 2030 SDGs (see https://sustainabledevelopment.un.org) provide a framework for humanity and the planet for today and tomorrow. At its core are 17 SDGs that represent calls for action by all countries. The SDGs are underpinned by the environment—that is, maintaining ecosystem health (Goal 13: combat climate change and its impacts; Goal 14: life below water; Goal 15: life on land) is a necessary precondition to achieving the 2030 agenda (Lynch *et al*., 2017; Reid *et al*., 2017). The scientific community has much to contribute toward achieving the SDGs, and conservation physiology is no exception (see Table 1). The SDGs have a series of targets and indicators. For example, target 15.5 is ‘Take urgent and significant action to reduce the degradation of natural habitats, halt the loss of biodiversity and, by 2020, protect and prevent the extinction of threatened species’. To achieve this target (and keep organisms off the IUCN Red List or lower their listing status—i.e. the indicator), it is necessary to understand the drivers of population declines. Conservation physiology is well positioned to identify the specific drivers of decline (i.e. the stressors) as well as their interactions, which can be additive (or synergistic), neutral or subtractive (antagonistic). In doing so, we can identify potential opportunities for mitigating such stressors. Otherwise, valuable resources could be devoted toward management options that fail to address the cause of the declines. For example, although much science points toward dams and other forms of physical habitat alteration contributing to population declines of Pacific salmon (*Oncorhynchus* spp.; Lackey 1999), recent experimental physiology studies have revealed that atypically warm water also constrains migration success and can kill salmon (Martins *et al*., 2012). If recovery efforts focused solely on dam removal and physical habitat restoration, we could lose the opportunity to create in-river thermal refuges and to limit fishing effort during the warmest periods. Quite simply, one needs to know the driver(s) of the problem to apply the appropriate intervention. Physiological studies can implicate cause-and-effect relationships that reveal mechanistic pathways and provide certainty for regulators (Cooke and O’Connor, 2010) as well as the basis for extrapolation and visualization via modelling approaches (Deutsch *et al*., 2015; Dahlke *et al*., 2018). Ultimately, implementation of SDGs is hindered by a conflict between biodiversity conservation and economic development (Scherer *et al*., 2018). Goal 2 (zero hunger, food security) can help diffuse such a tension, as maintaining adequate food supplies is important to everyone, regardless of politics and food production depends on biodiversity, especially under impending climate change scenarios (de la Barrera and Andrade, 2005). Physiological tools are well suited to help test local varieties and wild relatives of crops and in developing sustainable agroecological practices.

## Synthesis and realizing success in conservation physiology

There is growing appreciation for the multitude of ways in which physiological data can supplement conventional conservation approaches. In this paper, we have identified several conservation opportunities where physiological tools and experimental approaches could augment existing efforts to stem and reverse the global loss of biodiversity. Although the specific topics covered here are not necessarily new conservation issues, they reflect the broad range of challenges facing conservation biology practitioners and highlight the considerable potential for conservation physiology to contribute to identifying and managing current and emerging threats. Given the diverse range of issues facing conservation science, realizing the potential for physiology to contribute to the management and recovery of biodiversity loss requires an appreciation of the equally diverse ways that physiological systems respond to environmental change (Madliger and Love, 2015). Moreover, by integrating physiological metrics into conventional approaches, we can improve the uptake of evidence-based decision-making in conservation (Cooke and O’Connor, 2010; Cooke, 2014; Coristine *et al*., 2014; Cooke *et al*., 2017).

Robust decision-making for conservation requires a sound understanding of cause–effect relationships between organisms and environmental stressors and between management interventions and ecological outcomes (Coristine *et al*., 2014; Cooke *et al*., 2017; Nichols *et al*., 2017). Conservation physiology differs from many conventional conservation approaches in that it can clearly demonstrate the necessary causal relationships between stressors and organismal responses (Carey, 2005; Wikelski and Cooke, 2006; Cooke and O’Connor, 2010). Moreover, because physiology is inherently grounded in mechanisms, physiological responses explicitly define the nature of the relationship between organisms and their stressors. These mechanistic underpinnings also provide considerable predictive power—known responses to stressors can be used to model predicted impacts of a threat which can facilitate preparedness, or better yet, can direct and inform threat abatement strategies (Coristine *et al*., 2014; Cooke *et al*., 2017). This is particularly vital given the myriad interacting stressors acting on species and systems and the need to tease apart the threats and triage such strategies. Conservation physiology can be used to identify traits that may pre-determine an individual’s or a population’s suitability for conservation management or to identify specific environments that provide critical refuge from stressors (Wikelski and Cooke, 2006). Identifying which trait or traits may be the best metric for identifying an impact is likely to be organism-, context- and challenge-specific (MacMillan, 2019), but through experimentally rigorous testing and systematic reviews of existing literature and by monitoring a diverse panel of physiological markers, these difficulties can be lessened and contribute to building a stronger evidence base for conservation actions.

Emerging technologies have the potential to bring considerable change to the way in which physiological tools are applied to conservation issues by integrating physiological responses over a range of temporal, spatial and biological scales. Although not the focus of this review (see Madliger *et al*., [2018] for recent review of the evolving conservation physiology toolbox), the tools themselves are what enable our community to increasingly be relevant to policymakers and practitioners—often in near real time. For example, technologies are already widely used to remotely monitor physiological parameters in free-living animals, to identify and locate organisms and assess the status of their habitats (Pimm *et al*., 2015). This rapidly developing field is likely to expand the capacity of conservation practitioners to apply physiology tools to a wider range of species with an increased range of integrated sensors, better data transfer technology and at an ever-decreasing size and cost. For example, biotelemetry and bio-logging are increasingly being utilized for status assessment and in the design of effective protected areas for the management of endangered species (Fraser *et al*., 2018; Dwyer *et al*. 2019) and to assess climate change impacts (Chmura *et al*., 2018), in part due to technological advances that have minimized the invasiveness of tags on animals. Increasing access to global monitoring technologies is also facilitating better assessment of emerging environmental changes which can allow us to target studies to precede or track impacts in real time (He *et al*., 2015). Increasing spatial resolution of satellite data, the availability of multispectral and hyperspectral sensors for remote and large-scale vegetation condition assessments (He *et al*., 2015), and drones are increasing the capacity to remotely collect and distribute data (Pimm *et al*., 2015; Malenovsky *et al*., 2017). Likewise, access to and the affordability of cutting edge molecular tools is allowing genomic technologies (e.g. environmental DNA, microbiome, genome and transcriptome sequencing) to be integrated into traditional conservation approaches where it can contribute to a more holistic and integrated picture of organism functioning (Taylor and Gemmell, 2016).

Although numerous opportunities exist for conservation physiology to make significant advancements to the way environmental management and ecosystem restoration is conducted, the challenge remains to ensure that our science is relevant and high quality, to ensure the messaging is straightforward and accessible to end users and to ensure that a diverse range of voices are represented. To this end, the journal *Conservation Physiology* provides a forum for the open access publication of conservation-relevant physiological research. However, publication biases in conservation science, whereby the majority of current conservation research does not occur on the most threatened organisms or in the most biodiverse regions (Wilson *et al*., 2016), impairs our ability to protect and manage the ecosystem services on which humans depend. Emerging prospects in conservation physiology could provide researchers from low- to middle-income countries with additional opportunities to contribute to practical conservation in areas where this research is most needed.

There is growing awareness regarding the importance of traditional knowledge and Indigenous engagement in biodiversity research and protection (Cvitanovic *et al*., 2015; Garnett *et al*., 2018). More than one quarter of all lands outside of Antarctica are owned and/or managed by Indigenous people, making their relative contribution to meeting global biodiversity frameworks like the Convention on Biological Diversity, of critical importance (Garnett *et al*., 2018). Recognizing and respecting the enormous value of Indigenous biocultural heritage for ecosystem protection and management is vital to inform conservation efforts both within and outside of Indigenous-managed lands. By acknowledging traditional and local knowledge of both historical and current conditions, conservation physiology can better understand the capacity of organisms and ecosystems to respond to current environmental challenges (Cvitanovic *et al*., 2015). There are still relatively few examples of where this has occurred in practice so this is an area of great opportunity. Similarly, crowd-sourced citizen science increases public engagement with conservation activities (McKinley *et al*., 2017; Yang *et al*., 2019) and potentially provides a useful way to collect and integrate coarse physiological metrics such as growth, condition indices and reproductive status into conservation actions. The development of smartphone apps (Andrachuk *et al.*, 2019) and freely available image analyses tools (e.g. Google Image) is promoting the value of citizen science for conservation physiology by creating a network of integrated environmental sensors, providing new ways of streamlining data collection and management and expanding the pool of data collectors and analysers (Pimm *et al.*, 2015; McKinley *et al.*, 2017; Yang *et al.*, 2019). Overall, the horizon scan we have presented provides evidence that, as the discipline of conservation physiology continues to mature, we have numerous opportunities to promote integration, inclusivity and forward-thinking goals.

## Conclusion

To conclude, we are hopeful that conservation physiology will continue to deliver on its promise to not only document conservation problems but also help achieve solutions (Madliger *et al*., 2016). Here, we highlighted a number of promising and forward-looking ways in which conservation physiology has much opportunity to be relevant to the myriad of environmental and conservation challenges that are pervasive in the Anthropocene. Yet, we also recognize that conservation physiology alone is insufficient and will be most effective when conservation physiologists collaborate with those working in other scientific domains. Moreover, partnership with practitioners and policymakers will be essential to ensure that the work of the conservation physiologist is relevant and actionable. There are also a number of emerging opportunities to involve broader communities (e.g. stakeholders) in conservation physiology and to respectfully combine different forms of knowledge (including Indigenous knowledge). Our horizon scan provides evidence that, as the discipline of conservation physiology continues to mature, it provides a wealth of opportunities to promote integration, inclusivity and forward-thinking goals that contribute to achieving conservation gains and solving environmental problems. Conservation physiologists must ensure that their science is relevant with accompanying messaging that is straightforward and accessible to end users.
